# Special Issue “Dendritic Cell and Cancer Therapy 2.0”

**DOI:** 10.3390/ijms26199354

**Published:** 2025-09-25

**Authors:** Serena Zanotta, Domenico Galati

**Affiliations:** Hematology-Oncology and Stem Cell Transplantation Unit, Department of Hematology and Innovative Diagnostic, Istituto Nazionale Tumori-IRCCS-Fondazione “G. Pascale”, 80131 Napoli, Italy; s.zanotta@istitutotumori.na.it

Cancer continues to represent a major global health challenge, accounting for more than 9 million deaths in 2020. While chemotherapy, radiotherapy, and surgery remain the pillars of treatment, their toxicity profiles and limited long-term efficacy have accelerated the search for innovative therapeutic strategies. Among these, dendritic cell (DC) vaccination has emerged as a promising approach to mobilize host immunity against tumors [1,2,3,4].

The rationale lies in DC biology. In many pathological contexts, including cancer, DCs are numerically and functionally impaired. Yet, when fully matured, they orchestrate adaptive immunity by processing and presenting tumor-associated antigens (TAAs) to T lymphocytes, thereby initiating effector immune responses [5,6,7]. Unfortunately, within the tumor microenvironment (TME), chronic immunosuppression hampers DCs maturation and promotes tolerance, undermining effective antitumor activity [5]. Successful DC-based vaccines must therefore combine efficient TAAs presentation with robust co-stimulatory signals, ensuring activation of both T cells and natural killer (NK) cells [5,6,7,8].

Thanks to methodological advances, these biological insights have translated into more sophisticated clinical protocols, positioning DC vaccination at the cutting edge of modern cancer immunotherapy [1,3,7].

This second edition of the Special Issue highlights such progress, emphasizing how diverse strategies converge to enhance DC function and therapeutic efficacy.

One of the major hurdles in DC-based immunotherapy lies in achieving efficient antigen uptake and presentation. To address this, VP-R8, a D-octa-arginine polymer, was developed as an antigen-delivery and adjuvant system. In a murine T-cell lymphoma model, VP-R8 outperformed the conventional carrier keyhole limpet hemocyanin (KLH) by significantly increasing antigen capture by DC2.4 cells, promoting maturation (major histocompatibility complex class I/II [MHC-I/II], CD80, CD86 upregulation), and enhancing cell viability. In vivo, vaccination with VP-R8–conditioned, EL4-lysate-pulsed DCs suppressed tumor growth and enriched CD8^+^ T cells in the TME, with evidence of heightened cytotoxic activity (CD107a^+^). Splenocytes from treated mice produced more interferon-gamma (IFN-γ) and displayed stronger cytotoxic T lymphocyte (CTL) responses against EL4 targets. These findings support VP-R8 as a potent enhancer of DC-based vaccination, particularly in T-cell lymphomas, though validation in human-relevant models remains essential [9].

While delivery platforms like VP-R8 strengthen antigen uptake, the ultimate success of DCs therapy also hinges on how these cells mature and function within the tumor and its draining lymph nodes.

The ability of DCs to mount antitumor immunity is not uniform across contexts, as highlighted by studies in laryngeal squamous cell carcinoma. Analysis of 73 patients revealed that S100^+^ DCs, including mature forms, were abundant in healthy lymph nodes but markedly reduced in metastatic nodes and tumors. Importantly, higher S100^+^ DC levels in metastatic nodes correlated with improved survival, whereas a predominance of intratumoral CD1a^+^ immature DCs predicted poorer outcomes. Following radio/chemotherapy (RT/CRT), DC numbers declined in tumors and metastatic nodes but increased in unaffected healthy nodes, suggesting that RT/CRT impairs DC activity in irradiated tumor regions while leaving immune surveillance intact elsewhere [10].

This study underscores a crucial concept: beyond antigen delivery, the tumor environment profoundly dictates the fate and function of DCs. This principle becomes especially evident in breast cancer, where chemotherapy reshapes DC subsets in ways that directly affect therapeutic response.

An observational study in breast cancer patients treated with neoadjuvant chemotherapy (NAC) demonstrated that intratumoral plasmacytoid dendritic cells (pDCs) play a decisive role in therapy response. High densities of CD123^+^ pDCs correlated with greater residual cancer burden, lack of pathological complete response (pCR), higher grade and stage, and human epidermal growth factor receptor 2 (HER2)-negativity. Remarkably, all tumors achieving pCR contained <20 CD123^+^ pDCs/mm^2^, and each additional pDC/mm^2^ reduced the odds of pCR by ~13%. In contrast, associations with DC-LAMP^+^ DCs were inconsistent, and CD1a^+^/DC-SIGN^+^ DCs offered limited predictive value [11].

Taken together, these observations position pDCs not only as biomarkers of treatment response but also as actionable targets. This duality has inspired efforts to reprogram their function therapeutically, leading to the development of innovative immunotherapeutic strategies.

Plasmacytoid DCs are potent type-I interferon (IFN-I) producers that bridge innate and adaptive immunity. However, within the TME, tumor-derived cytokines and regulatory T cells can shift them toward a tolerogenic, tumor-promoting phenotype. Conversely, when properly activated, pDCs can exert direct tumoricidal effects via tumor necrosis factor-related apoptosis-inducing ligand (TRAIL) and granzyme B, and prime cytotoxic T cells. Therapeutic approaches under exploration include in situ activation with toll-like receptor (TLR) 7/9 agonists (e.g., imiquimod, CpG) and adoptive pDC vaccination. Early trials using autologous or allogeneic pDCs have shown safety and induction of tumor-specific T cells, though limitations such as low abundance of circulating pDCs and tumor-mediated suppression persist. Combining pDC-based approaches with immune checkpoint inhibitors (ICIs) or genetic engineering strategies may overcome these barriers [12].

Capitalizing on these insights, researchers have turned to allogeneic pDC platforms as a way to overcome the logistical and biological challenges of autologous vaccines.

To address the variability and scalability issues of autologous DC-based vaccines, the allogeneic pDC platform PDCline was tested in non-small cell lung cancer (NSCLC). In peripheral blood mononuclear cells (PBMCs) from 26 patients, 85% mounted responses to at least one of 14 peptides, and 69% to at least two. Strong reactivity was observed against melanoma-associated antigen A2 (MAGE-A2), melanoma-associated antigen A9 (MAGE-A9), and survivin. Importantly, combining PDCline with pembrolizumab expanded both response breadth and magnitude. Notably, antigen reactivity was independent of tumor histology and not strictly correlated with antigen expression, supporting its broad applicability [13].

This off-the-shelf model marks a significant advance toward integrating pDC vaccination with ICIs, underscoring the translational momentum in this field. Paradoxically, however, the very same lineage that offers therapeutic promise can also give rise to malignancy, exemplified by Blastic Plasmacytoid Dendritic Cell Neoplasm (BPDCN).

BPDCN is a rare and aggressive neoplasm, predominantly affecting older men, with historically poor outcomes under conventional chemotherapy or stem-cell transplantation. The discovery of near-universal CD123 overexpression has reshaped treatment paradigms. Tagraxofusp, an interleukin-3 (IL-3)–diphtheria toxin fusion protein, has demonstrated significant efficacy but carries risks of capillary-leak syndrome. More recently, the anti-CD123 antibody–drug conjugate IMGN632 has shown encouraging efficacy with an improved safety profile. In addition, CD123-targeted chimeric antigen receptor T cells (CAR-T cells) and bispecific T-cell engagers (e.g., Flotetuzumab, APVO436) are in clinical development. Importantly, resistance to Tagraxofusp often arises from defects in the diphthamide-biosynthesis pathway rather than loss of CD123, providing a rationale for combination strategies with epigenetic agents such as azacytidine or the B-cell lymphoma 2 (BCL-2) inhibitor Venetoclax [14].

Together, these insights illustrate a recurring theme: whether as therapeutic allies or malignant adversaries, DCs remain central to shaping cancer outcomes and guiding precision immunotherapy strategies.

Collectively, these studies reaffirm DCs as central orchestrators of antitumor immunity and strengthen the case for DCs vaccination as a viable therapeutic strategy. Innovations such as VP-R8–mediated antigen delivery and scalable allogeneic pDC platforms enhance antigen uptake, maturation, and T-cell priming, while combinations with ICIs broaden response potential. Intratumoral biomarkers, particularly CD123^+^ pDC density following NAC, emerge as powerful tools for patient stratification and rational trial design. In parallel, malignant pDCs in BPDCN exemplify how mechanistic insights drive therapeutic breakthroughs through CD123 targeting.

Future priorities include harmonizing clinical endpoints, standardizing manufacturing, and embedding biomarker-driven randomized trials to translate immunogenicity into durable survival benefits. DCs remain at the intersection of innovation, challenge, and promise, serving both as therapeutic allies and as pathological adversaries, yet always at the core of advancing cancer immunotherapy.

## Data Availability

Not applicable.
